# Editorial Comment: Health-Related Quality of Life in Hemodialysis Patients: An Iranian Multi-Center Study

**DOI:** 10.5812/numonthly.16986

**Published:** 2014-03-09

**Authors:** Ehsan Nobakht, Scott D Cohen

**Affiliations:** 1 Division of Renal Diseases and Hypertension, George Washington University, Washington, DC, USA

**Keywords:** Quality of Life, Kidney Failure, Chronic, Renal Dialysis

*Dear Editor,*

Psychosocial aspects of health have a significant impact on the clinical outcomes of patients with end stage renal disease (ESRD) on hemodialysis (HD) (1, 2). Quality of life (QOL) is a potentially modifiable risk factor for mortality in this patient population. Mapes et al. (3) previously showed that lower health related quality of life (HRQOL) scores are associated with higher hospitalizations and increased mortality in dialysis patients. The mental and physical component summary (MCS, PCS) scores and kidney disease composite summary (KDCS) score are frequently used to assess HRQOL throughout the world. However, there is limited data on the QOL of ESRD patients from the Middle East. 

Rostami et al. (4) recently performed a cross-sectional study designed to evaluate the perception of QOL in 6,930 chronic HD patients from Iran. This study is important, as it is the largest comprehensive data collection addressing HRQOL of HD patients in Iran. An Iranian adapted version of the kidney disease QOL short form version 1.3 (KDCS-SF1.3) questionnaire was used. This instrument was previously validated in general medical patients in Iran (5). They compared PCS, MCS and KDCS scores of their patients with 19 similar studies performed in America, Europe and Asia between 1996 and 2010. Patients with acute illnesses requiring hospitalization and vascular access failure requiring temporary catheter placement were excluded from the study. PCS and MCS scores were slightly higher than the overall results while KDCS was slightly lower than the overall results. 

A significant limitation of the study is that the majority of patients had a low literacy rate which could potentially skew the results of this questionnaire. The Iranian adapted version of the questionnaire was previously validated in healthy individuals in Tehran which may have higher literacy rate than the rest of the country (5). The study also did not have a control group of general medical patients to compare the results of the QOL scores. 

Dialysis adequacy as defined by a Kt/V between 1 and 1.2 was associated with a lower rate of hospitalization. As with many clinical variables, there is also a J-shaped relationship between URR and survival in HD patients (6). Adverse outcomes observed among patients with a higher URR and KT/V may reflect lower body mass and malnutrition which should prompt a nutritional status evaluation (7).

Despite potential differences in culture and perceptions of QOL, this study suggests that the QOL of Iranian HD patients are similar to that of ESRD patients from other countries. Prospective studies are needed to better understand the impact of HD on the QOL of ESRD patients. Interventions to improve the QOL of ESRD patients around the world are urgently needed.

## References

[1] Cohen SD, Kimmel PL (2013). Quality of life and mental health related to timing, frequency and dose of hemodialysis.. Semin Dial..

[2] Kimmel PL, Cohen SD, Weisbord SD (2008). Quality of life in patients with end-stage renal disease treated with hemodialysis: survival is not enough!. J Nephrol..

[3] Mapes DL, Lopes AA, Satayathum S, McCullough KP, Goodkin DA, Locatelli F (2003). Health-related quality of life as a predictor of mortality and hospitalization: the Dialysis Outcomes and Practice Patterns Study (DOPPS).. Kidney Int..

[4] Rostami Z, Einollahi B, Lessan-Pezeshki M, Soleimani Najaf Abadi A, Mohammadi Kebar S, Shahbazian H (2013). Health-related quality of life in hemodialysis patients: an Iranian multi-center study.. Nephrourol Mon..

[5] Montazeri A, Goshtasebi A, Vahdaninia M, Gandek B (2005). The Short Form Health Survey (SF-36): translation and validation study of the Iranian version.. Qual Life Res..

[6] McClellan WM, Soucie JM, Flanders WD (1998). Mortality in end-stage renal disease is associated with facility-to-facility differences in adequacy of hemodialysis.. J Am Soc Nephrol..

[7] Chertow GM, Owen WF, Lazarus JM, Lew NL, Lowrie EG (1999). Exploring the reverse J-shaped curve between urea reduction ratio and mortality.. Kidney Int..

